# Nano-Osmolyte Conjugation: Tailoring the Osmolyte-Protein Interactions at the Nanoscale

**DOI:** 10.1021/acsomega.3c07248

**Published:** 2023-11-16

**Authors:** Hemlata Sharma, Tanveer Ali Dar, Yasanandana Supunsiri Wijayasinghe, Dibakar Sahoo, Nitesh Kumar Poddar

**Affiliations:** † Department of Biosciences, Manipal University Jaipur, Jaipur-Ajmer Express Highway, Dehmi Kalan, Near GVK Toll Plaza, Jaipur, Rajasthan 303007, India; ‡ Department of Clinical Biochemistry, University of Kashmir, Srinagar 190006, Jammu and Kashmir India; § Department of Biochemistry and Clinical Chemistry, Faculty of Medicine, University of Kelaniya, Ragama 11600, Sri Lanka; ∥ School of Physics, Sambalpur University, Jyoti Vihar, Burla 768019, Odisha, India

## Abstract

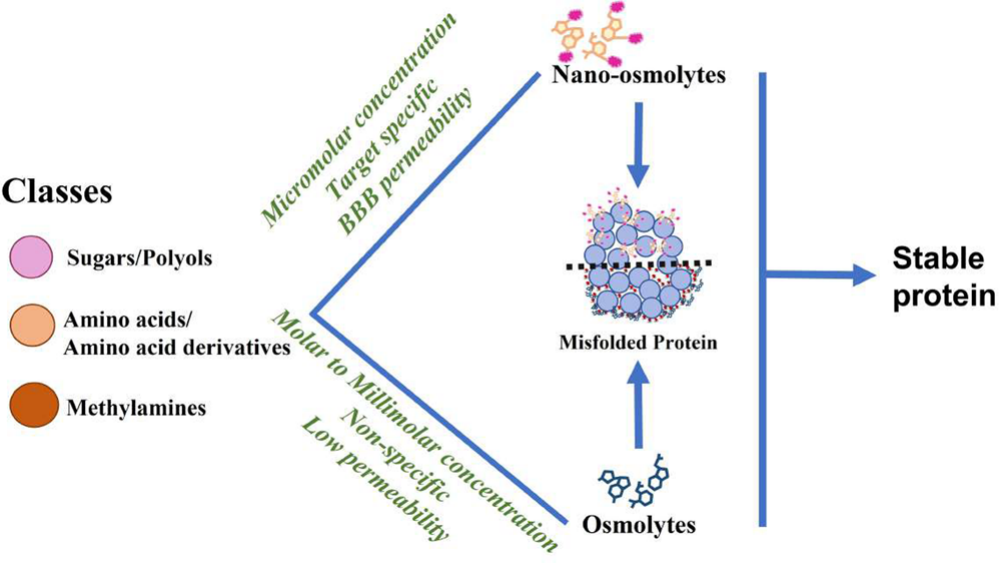

Osmolytes are small organic compounds accumulated at higher concentrations in the cell under various stress conditions like high temperature, high salt, high pressure, etc. Osmolytes mainly include four major classes of compounds including sugars, polyols, methylamines, and amino acids and their derivatives. In addition to their ability to maintain protein stability and folding, these osmolytes, also termed as chemical chaperones, can prevent protein misfolding and aggregation. Although being efficient protein folders and stabilizers, these osmolytes exhibit certain unavoidable limitations such as nearly molar concentrations of osmolytes being required for their effect, which is quite difficult to achieve inside a cell or in the extracellular matrix due to nonspecificity and limited permeability of the blood–brain barrier system and reduced bioavailability. These limitations can be overcome to a certain extent by using smart delivery platforms for the targeted delivery of osmolytes to the site of action. In this context, osmolyte-functionalized nanoparticles, termed nano-osmolytes, enhance the protein stabilization and chaperone efficiency of osmolytes up to 10^5^ times in certain cases. For example, sugars, polyols, and amino acid functionalized based nano-osmolytes have shown tremendous potential in preventing protein aggregation. The enhanced potential of nano-osmolytes can be attributed to their high specificity at low concentrations, high tunability, amphiphilicity, multivalent complex formation, and efficient drug delivery system. Keeping in view the promising potential of nano-osmolytes conjugation in tailoring the osmolyte–protein interactions, as compared to their molecular forms, the present review summarizes the recent advancements of the nano-osmolytes that enhance the protein stability/folding efficiency and ability to act as artificial chaperones with increased potential to prevent protein misfolding disorders. Some of the potential nano-osmolyte aggregation inhibitors have been highlighted for large-scale screening with future applications in aggregation disorders. The synthesis of nano-osmolytes by numerous approaches and future perspectives are also highlighted.

## 1 Introduction

Osmolytes are low-molecular-weight naturally occurring organic compounds in living organisms with an ability to maintain cell volume and protect cells during stress conditions such as high temperature, high pressure, and high salinity. These osmolytes can be categorized into five groups, namely sugars (e.g., sucrose), polyols (e.g., glycerol, mannitol, inositol), amino acids (e.g., proline, taurine), methylamines (e.g., trimethylamine *N*-oxide), and urea.^1−3^ All osmolytes, except urea, can stabilize cellular proteins, thus preserving cellular functionality under unfavorable conditions. Among other features, two main attributes of these osmolytes for their evolutionary selection as biological protectants include the ability to provide immense stability to macromolecules under denaturing conditions and compatibility with cellular functions.^4^ Moreover, osmolytes like betaine and trimethylamine *N*-oxide (TMAO) exhibit therapeutic potential against several human diseases and also act as disease markers in certain cases.^5^ Additionally, some osmolytes play a vital role in regulating the key pathways of amyloid formation and modulating the aggregation of several disease-causing proteins associated with neurodegenerative disorders including Alzheimer’s and Parkinson’s diseases.^5^ Owing to this, osmolytes have been termed as chemical chaperones with the ability to aid in protein folding. Although very promising, osmolytes do have some limitations in exploiting their chaperone activity.^6^ For example, nearly molar concentrations of the osmolytes are required for their biological effect, which is difficult to achieve within the cell or in extracellular space.^7−9^ Moreover, being highly hydrophilic, osmolytes readily distribute throughout the body, which dramatically reduces their effective concentration. Osmolytes such as taurine, myo-inositol, and glucose fail to transport the blood–brain barrier (BBB) under different stress conditions and represent the chronic condition due to their limited availability in the brain cells.^10−15^ In addition, the impermeability of the BBB to osmolytes further limits their applications in neurological diseases.^10−15^

In light of these limitations, nanoconjugation of these small-molecular-weight osmolytes is emerging as a potential strategy for not only enhancing their efficiency as protein folders but also modulating their bioavailability with enhanced specificity. Several studies have come up wherein nanoencapsulation of osmolytes endows them with varied beneficial properties such as high specificity, biocompatibility, biodegradability, water solubility, and low toxicity.^13,14,16^ Moreover, nanocarrier systems can be exploited as an emerging delivery method to enhance the permeability of the BBB through various strategies such as PEGlylated liposomes, functionalized nanoparticles, and microcapsules and thereby releasing the therapeutic amount of nano-osmolytes with prolonged shelf life to the targeted site of the brain.^17^ Recently, researchers have shown that polylactide-*co*-glycolide (PLGA) NP conjugated glycopeptide can pass the BBB at the optimum level as a reference to the injected dose.^18^ In this direction, PEGylated conjugated biomolecules or polymeric nanoparticles have been extensively used as nanocarriers against the disadvantages of using nanoparticles in terms of immunogenicity, cytotoxicity, drug leakage, reticuloendothelial system uptake, and hemolysis.^17^ Therefore, the design of nano-osmolyte drugs that are nontoxic, biocompatible, and target-specific is an urgent need for better therapeutics of protein aggregation disorders.^19,20^

Keeping in view the importance and emergence of nano-osmolyte conjugates as future therapeutic agents, the present review, for the first time, was designed to provide an overview of the current status of the nano-osmolyte conjugation as a promising strategy for tailoring osmolyte–protein interaction for enhanced chaperonic effects and efficient prevention against protein aggregation. Moroever, the present review might aid in the identification of some novel nano-osmolyte conjugates with immense therapeutic potential against protein misfolding diseases. Future insights in this direction have also been highlighted.

## 2 Osmolytes as Efficient Protein Folders and Aggregation Modulators

**Figure 1:**
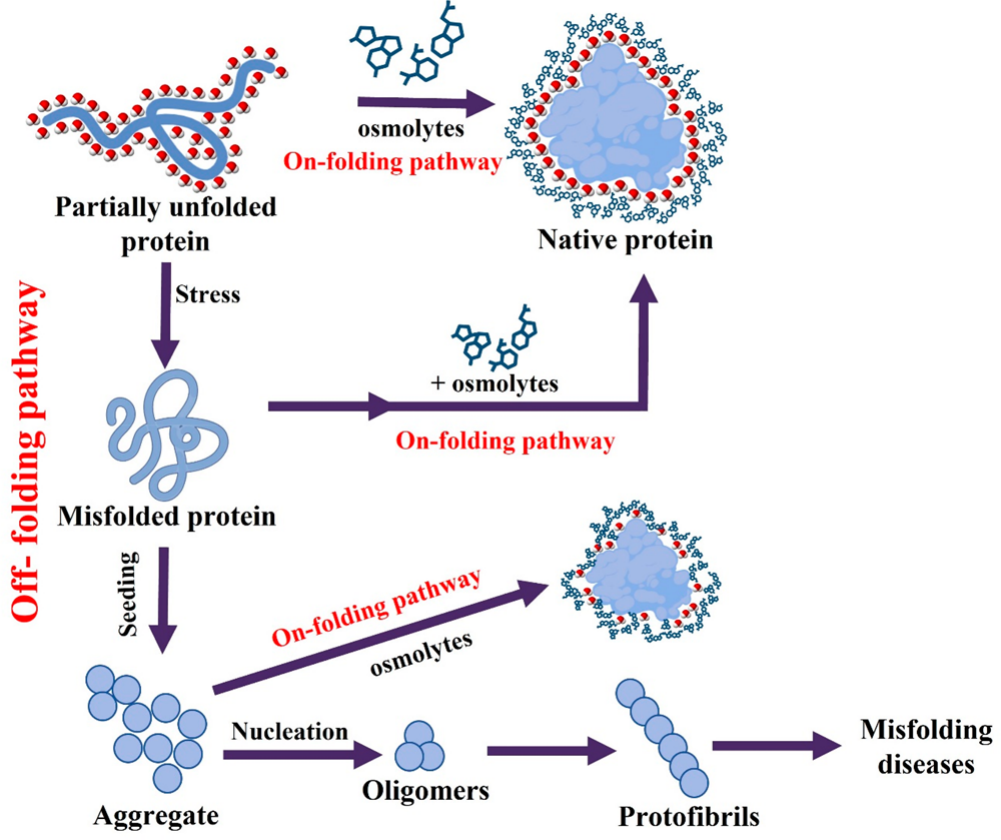
On-/off-folding pathways of protein folding through a seeding/nucleation mechanism and stabilization of protein folding by osmolytes.

Proteins are linear assemblies of amino acids in a specific order which undergo folding into a unique three-dimensional structure resulting in a biologically active native form.^21^ Protein folding is a complex process due to the existence of so many possible conformations, out of which only one conformation governs its lowest energy native form.^21^ During folding, proteins move through different intermediate states in the energy landscape to achieve the most favorable lowest energy conformation as the native form. However, stress conditions such as extreme temperature, pH, high salt, and chemicals like urea may alter the three-dimensional structure of the proteins, resulting in protein denaturation or protein misfolding. Proper folding of the newly synthesized proteins with the help of molecular chaperones and chemical chaperones/osmolytes is highly beneficial in preventing protein aggregates. Like molecular chaperones which control the cellular dynamics of proteostasis conditions, chemical chaperones also accumulate in the cells at high concentrations to assist the folding of disaggregated proteins under different stress conditions (Figure 1).^22^ It has been reported that chemical chaperones influence the dynamics of proteome conditions of cells toward a more native and catalytic form.^23^

Generally osmolytes, also called chemical chaperones, are categorized as compatible and noncompatible osmolytes. Compatible osmolytes, being osmoprotective, change the equilibrium concentration of the unfolded state of the protein toward a more native conformation, whereas the noncompatible osmolytes, such as urea, perturb the structure of the protein change into an unfolded state with a more unfavorable interaction at the protein hydrophobic sites (Figure 1). On the basis of a preferential hydration model, the compatible osmolytes are preferentially excluded from the protein surfaces as being thermodynamically unfavorable toward the protein backbone. On the other hand, noncompatible osmolytes perturb the protein structure through their direct interaction with the protein backbone.^24,25^ It has been found that osmolytes such as proline and sorbitol interact more with the polar residues of the proteins in the elongation and fibrillation stages with respect to the native protein and prevent the protein from oligomerization to higher forms of aggregates.^26^

It has been found that the renal cells accumulate osmolytes such as inositol, glycerophosphorylcholine (GPC), and betaine under high interstitial NaCl and urea concentrations. Thus, these osmolytes counteract the harmful effects of urea on proteins in the renal cells. Similarly, a milieu of osmolytes is very much specific in the mutational buffering of various metastable protein intermediates to modulate the proteostasis-driving mechanism for the evolution of new protein functions.^27^ Nevertheless, it is important to note that some proteins (e.g., α-synuclein and prothymosin-α) remain disordered even in the native state.^28^ To enhance the *in vitro* stability and maintain the native states of the ordered proteins, stabilizers and osmolytes are added to the protein preparations.^29^ For example, a high concentration of TMAO induces the conformation of α-synuclein to a typical well-folded protein. However, most crowding agents cannot stabilize the unstructured forms of α-synuclein. It has been found that osmolytes like taurine and TMAO induce the folding structure of the casein protein, but taurine decreases the chaperone activity of the α-casein and β-casein beyond the physiological concentration.^27,30^ In a well-known molecular confinement experiment using an encapsulated protein within a silica matrix, most proteins showed higher thermal stability and were reversible to their native form. This reflects that denatured/unstructured protein can be directed to a native conformation in the presence of different cosolutes and molecular crowders.^31^ Thus, osmolytes play a vital role in modulating the folding kinetics of the proteins and will be useful in therapeutic interventions in protein-misfolding-associated diseases.

### 2.1 Role of Osmolytes in Modulating/Preventing Protein Misfolding/Aggregation

During the amyloid-like aggregation process, the protein native state converts into non-native states which are associated with elongated fibril formation followed by oligomerization of different sizes and thus the different structural species are influenced by the environment. This process can be influenced by the surrounding conditions: in particular, unfavorable environments may interfere with achieving the native conformation, and instead, proteins may be trapped in less stable partially or misfolded intermediates resulting in the aggregation of proteins. In some instances, the aggregated proteins cause human diseases such as Parkinson’s, Alzheimer’s, Huntington’s, and lysosomal storage diseases (LSDs).^32^ The process of amyloid or aggregated protein is based on a crystallization-like process popularly known as the seeding–nucleation model (Figure 1). This process consists of a long lag rate-determining phase followed by a rapid elongation step. In the lag phase, a thermodynamically unfavorable interaction involves misfolding protein and this seeding of unorganized oligomer formation leads to the rapid elongation of fibril formation and conversion into larger aggregates (Figure 1).^33,34^ Osmolytes play an important role in stabilizing the newly synthesized/misfolded protein through nonspecific differential interaction of backbone and side chain residues of the protein and thus prevent further aggregation of the protein as described earlier (Figure 1).

**Figure 2:**
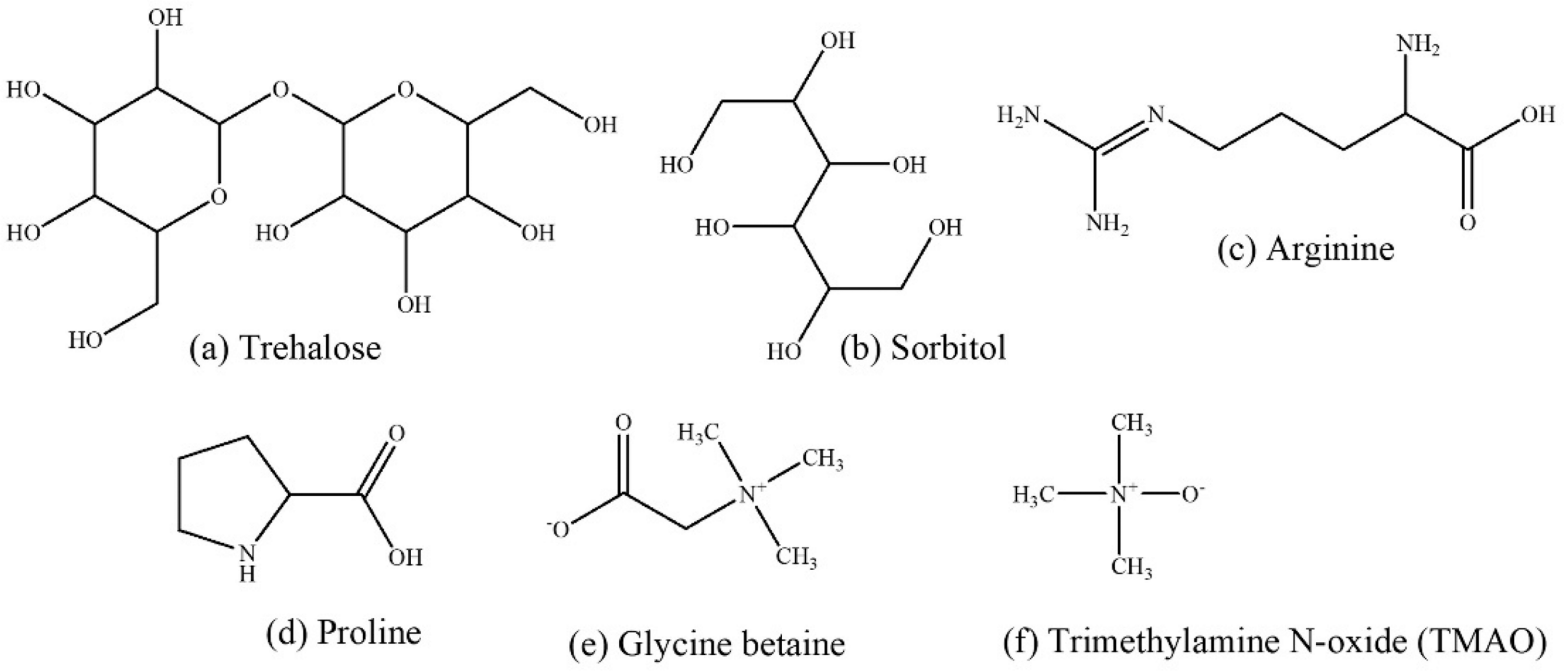
List of major osmolytes for the prevention of protein aggregation.

The different classes of osmolytes affect protein fibrillation and fibrillation kinetics in different ways.^35^ Proline prevents aggregation and accumulation of different types of proteins, such as lysozyme^36^ and P39A retinoic acid binding protein.^37^ Another example is α-synuclein, an intrinsically disordered proteins (IDP), wherein it has been reported that in the presence of 3M TMAO, α-synuclein is converted into heterogenous oligomers. Also, TMAO behaves differently in the presence of prion protein and forms oligomers.^38^ At the same time, TMAO prevents the transformation of the amyloidogenic form of prion protein *in vivo* system.^38^ In general, the β-helical secondary structure content increases while the α-helical content decreases in the protein during aggregation. This form of aggregation is attributed to a major implication of neurodegenerative diseases and cardiovascular and metabolic disorders.^39^ The amino acid l-proline is one of the osmolytes that works exceptionally well in inhibiting such aggregation. Proline aids in the regulation of intrinsically disordered proteins (IDPs).^40^ It has been observed that proline residues are significantly much higher in IDPs that favor more cis-pro populations and these populations create long-range contacts, resulting in the enrichment of specific features of conformational ensembles in the changing cellular environment.^41^ Proline is amphipathic with imino/carboxyl and methylene groups of the pyrrolidine rings that provide the polar and hydrophobic regions in the supramolecular assembly. In the event of protein aggregation, the hydrophobic surface of the proline interacts with the solvent-exposed hydrophobic patches of the protein and thus the protein aggregation is inhibited during the protein folding.^42^ Moreover, Ignatova et al. have shown that since proline contains charged groups, it might induce electrostatic repulsion on interacting with a polypeptide chain at the start of protein aggregation.^43^ For example, proline at higher concentrations has been reported to encourage folding and inhibit aggregation of bovine carbonic anhydrase and lysozyme.^39,44^ Among many osmolytes, trehalose, sorbitol, arginine, glycine-betaine, TMAO, and proline are industrially important osmolytes that prevent the protein misfolding from aggregation (Figure 2).^45^ It has been found that trehalose, sorbitol, and arginine are effective excipients for the formulation and purification of therapeutic proteins.^29,46^ Many neurological diseases are associated with the progressive accumulation of misfolded or aggregated proteins in the brain.^47^ Huntington’s disease is a neurodegenerative disorder caused by the accumulation of polyglutamine-rich “huntingtin” protein in the neuronal nucleus.^48^ Tanaka and colleagues reported that oral administration of trehalose effectively reduced the huntingtin protein accumulation, which was a breakthrough discovery of trehalose as a therapeutic agent against Huntington’s disease.^49^ Moreover, sucrose and trehalose were effective in inhibiting amyloid-beta aggregation. These osmolyte supplementations were also shown to promote neuroprotection in Alzheimer’s by inducing autophagy.^50^ Four osmolytes, namely betaine, raffinose, sarcosine, and taurine, are effective in the disaggregation and inhibition of the TGFBI protein. Corneal dystrophies are TGFBI (transforming growth factor beta-induced) protein-associated disorders caused primarily by TGFBI protein aggregation in the cornea, which may lead to blindness in severe cases.^51,52^

In addition, osmolytes protect vaccines in terms of their formulation and stability. Trehalose protects hemagglutinin present in influenza vaccines from various stresses during vaccine production and storage.^53^ Moreover, since therapeutic monoclonal antibodies (mAbs) are prone to aggregation, osmolytes, namely betaine, sarcosine, ectoine, and hydroxyectoine, have been investigated for their ability to prevent the aggregation of IgG1 antibodies. Sarcosine and hydroxyectoine were found to increase the melting temperatures of the IgG1 mAbs, and sarcosine was found to be the most stabilizing osmolyte.^21^

## 3 Limitations of Osmolytes

Although the osmolytes are effective in stabilizing proteins or inhibiting protein aggregation, their effectiveness is limited at various levels, including endocytic cell uptake, blood–brain barrier crossing, and multivalent binding with aggregating proteins. In addition, higher molar concentrations of osmolytes under *in vitro* conditions are required to achieve their beneficial effects on protein stability and folding. However, the accumulation of molar concentrations under cellular conditions to execute their effects has detrimental effects on the cells. For example, a higher concentration of osmolytes influences the conformational stability of various intrinsically disordered transcription factors, promoting metastatic behavior of the cell.^54^ However, the interaction of osmolytes with proteins is weak and nonspecific and thus increased concentrations of the osmolytes are necessary to facilitate their interaction to ensure enhanced chaperone function. For instance, trehalose, being an osmolyte, activates the formation of a different oligomer of Aβ42. TMAO encourages tau fibrillation.^55^ Besides this, TMAO stimulates the fibrillar structure of S-carboxymethylated α-lactalbumin as well.^56^ In another study, glucose promotes the nucleation of Aβ42 and Aβ40, whereas galactose and mannose enhance the formation of mature fibrils. Studies reflect that different patterns of potential H-bonding would affect the process of fibrillogenesis.^57,58^

In light of this, increased attention is being diverted to overcoming these issues to harness the beneficial effects of the osmolytes in protein-misfolding-associated diseases. Among others, the nanoparticle-based approach is being viewed as a promising strategy wherein osmolyte-functionalized nanoparticles have been generated with increased efficiency, specificity, and bioavailability.

## 4 Nanoconjugation of Osmolytes

A pharmacological drug can be targeted to an affected site through conventional routes such as oral, intravenous, rectal, subcutaneous, intramuscular, transdermal, etc. to treat medical problems.^59^ However, this conventional approach to a drug delivery system has some limitations such as reduced bioavailability, undesirable side effects, frequent dosing, and unpleasant organoleptic properties.^60^ Nanoparticles can be engineered as nanoconjugates with various therapeutic molecules to make smart delivery platforms to the targeted region. Nanoconjugates are relatively less toxic than nanoparticles, do not cause mass poisoning, are less reactive, and do not lead to irreversible reactions with proteins in the body.^61^ One of the most attractive advantages of nanoconjugation is its ability to protect active pharmaceutical ingredients (APIs) from degradation. Moreover, nanoconjugated drugs can be tagged with fluorescent probes for imaging purposes to evaluate the therapeutic efficacy of drugs.^62−64^ In addition, single-cell nanoconjugation is a new field in cell-surface engineering, which is based on the protection of living cells against stress conditions.^65^

The nanobased conjugated approach has also been implemented in the preventive strategy for neurodegenerative diseases.^61,66^ These include the engineering of nanoparticles that will inhibit the process of protein fibril nucleation and the growth of protein fibrils and plaques.^67−70^ For example, many engineered nanoparticles such as hydrophobic polymer nanoparticles,^68^ quantum dots,^71,72^ carbon nanoparticles,^73^ and gold nanoparticles^69,74^ have been observed to inhibit protein aggregation. Peptide-coated gold nanoparticles^75^ and thioflavin-linked graphene oxide^76^ have been shown to inhibit the growth of fibrils in the presence of light. Nanoparticles have been designed with multiple amphipathic molecules to facilitate the interaction with the aggregated proteins.^77^ More importantly, nanoparticle-based systems have been able to cross the blood–brain barrier^78^ and target the amyloid fibril/plaque within the brain.^79^ These results suggest that nanoparticle-based platforms might be a good approach to inhibit the protein fibrillation process occurring in the human body.^77^ Similarly, nanoparticles can be employed to overcome the limitations of osmolytes and enhance the interaction of osmolytes with vulnerable proteins. However, suitable nanoparticles should be engineered to clear the misfolded and toxic protein plaques in the brain. The following section will illustrate various methods for the preparation of suitable nano-osmolytes.

## 5 Preparation of Nanoparticles and Nano-Osmolytes

Nanoparticles can be functionalized with osmolytes by several methods, which are described below. Iron-, zinc-, gold-, and silver-based nanoparticles are selected for this purpose due to their stability, biocompatibility, and relative safety in human applications.

### 5.1 Wet Chemical Method

Among different physical methods, a sol–gel is very efficient and popular. An important peculiarity of the sol–gel technology is the possibility to control the mechanism and kinetics of the chemical reactions, thus monitoring the materials’ final structure (particle size, porosity, thin-layer thickness). Typically, in sol–gel chemistry, an organometallic compound (an alkoxide, nitrate, or chloride) reacts under aqueous conditions to form a solid product. This product can be a dense glass monolith, a high-surface-area molecular filter, an aerogel to a metal oxide, a nitride coating, or a nanoparticle. It is a bottom-up synthesis method that begins with hydrolysis reactions, and a homogeneous sol is produced from precursors that condensed to form a gel, followed by aging, solvent extraction, and drying. It is an excellent method for various biomedical purposes due to the low processing temperature, inherent biocompatibility, synthesis of a mixture of nanoparticles, and environmental friendliness of the implied components. In this process, the primary homogeneous sol may consist of two or more types of the same size of nanoparticles and in one step the mixture of a sol is converted into a wet gel through a compaction process.^80^ This method can be used to synthesize many metal oxide nanoparticles such as ZnO, TiO_2_, SiO_2_, and ZrO_2_.^81^

### 5.2 Chemical Methods

These methods are simple, tractable, and efficient, in which the size, composition, and even shape of the nanoparticles can be controlled.^82^ Two chemical methods used for the synthesis of nano-osmolytes are as follows.

#### 5.2.1 Carbonization Method

Sugar-terminated nanoparticles are synthesized via carbonization of the molecular form of sugar/carbohydrates. The synthesis of sugar (e.g., glucose, maltose, and trehalose) nanoparticles involves heating an acidified aqueous solution of a molecular sugar at 90–100 °C.^82^ During the carbonization and nanoparticle formation, the colorless aqueous solution gradually turns brown within 1–2 h. Unreacted sugars are then removed by dialysis.

#### 5.2.2 Hydroxylation Method

The sugar (e.g., maltose) can be conjugated to carbon through the hydroxyl group. Trehalose can be conjugated into an iron oxide polymer.^83^ Pradhan et al. have reported the synthesis of nanoglutamine and nanoproline. At first, they synthesized polymer-coated γ-Fe_2_O_3_ nanoparticles. The γ-Fe_2_O_3_ nanoparticles were prepared by chemical methods. Briefly, a solution of octadecylamine, methylmorpholine *N*-oxide, and octadecene was mixed with constant stirring, followed by argon purging with heating. The resultant hydrophobic γ-Fe_2_O_3_ nanoparticles were separated by centrifugation and washing.^84^ For polyacrylate coating, the polymerization of hydrophobic γ-Fe_2_O_3_ nanoparticles was done by dissolving nanoparticles in reverse micelles, which produced an optically clear solution. Then nitrogen was purged into the optically clear solution. Finally, an ammonium persulfate solution was injected as a radical initiator for polymerization. The particles were washed with chloroform and ethanol and dissolved in distilled water. Polymer-coated nanoparticles were transformed into glutamine-conjugated nanoparticles by covalent linking. At first, the polymer-coated nanoparticle solution was mixed with a borate buffer of pH 9.0, a glutamine solution mixed with an ethanolic glutaraldehyde solution. After that, this mixture was added to a polymer-coated nanoparticle solution. Then the reduction was initiated in the imine bond formed by the reaction between aldehyde and amine by introducing an NaBH_4_ solution. Finally, nanoparticles were centrifuged and dialyzed. Similarly, proline-conjugated nanoparticles were prepared from polymer-coated nanoparticles by covalent linking between the nanoparticles’ primary amines and the proline’s carboxylic acid. Polymer-coated, amine-terminated nanoparticles were covalently conjugated with l-proline by an EDC:NHS reaction.

### 5.3 Green Synthesis and Biological Synthesis

Green synthesis and biological synthesis are two methods of synthesizing nanoparticles that have gained significant attention in recent years due to their potential for producing environmentally friendly and biocompatible nanoparticles. Green synthesis involves using plant extracts, microbes, or other natural sources as reducing agents and capping agents to synthesize nanoparticles.^85^ This method is considered green because it avoids the use of toxic chemicals. It is usually carried out at ambient temperature and pressure, reducing energy consumption and greenhouse gas emissions.^86^ Green synthesis has been successfully used to synthesize nanoparticles of various materials, including metals, metal oxides, and semiconductors, with different shapes, sizes, and properties.^87,88^ On the other hand, biological synthesis involves using microorganisms, such as bacteria, fungi, or algae, as living factories for synthesizing nanoparticles.^89^ Microorganisms are used to reduce metal ions or other precursors into nanoparticles using their metabolic pathways.^90^ This method is advantageous because it can produce nanoparticles with high yield and purity and can be easily scaled up for industrial production.^91^ Moreover, biological synthesis can also produce nanoparticles with unique properties that are difficult to obtain by other methods, such as protein coating or functionalization.^92^

In summary, green synthesis and biological synthesis are two distinct approaches to the synthesis of nanoparticles, both of which have unique advantages and limitations. Green synthesis mainly relies on plant extracts or other natural sources, whereas biological synthesis uses microorganisms as living factories. However, both methods offer significant potential for producing environmentally friendly and biocompatible nanoparticles.

## 6 Nano-Osmolytes and Protein Aggregation

**Figure 3:**
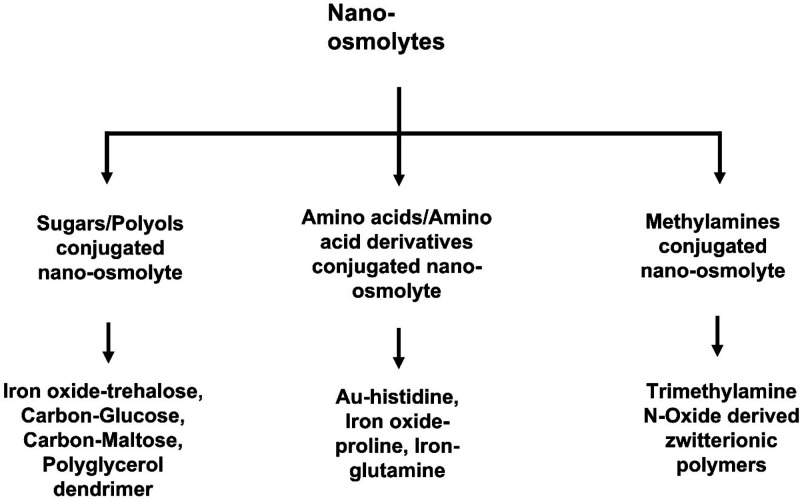
Proposed classification of nano-osmolytes.

Owing to the limitation of osmolytes in their molecular forms, increased attention has been diverted toward exploring the benefits of nanosizing the osmolytes vis-à-vis their effects on protein misfolding and aggregation. Based on this, several nanoconjugates of osmolytes have been evaluated for their ability to modulate protein aggregation. These nanoconjugated osmolytes can be classified into three main groups based on the class of osmolyte functionalized on the nanoparticles. These three classes include sugar/polyol nano-osmolytes, amino acid/amino acid derivative nano-osmolytes, and methylamine nano-osmolytes (Figure 3). The major details of the nano-osmolytes and their effects on protein aggregation have been summarized in Table 1. Some of the successful nano-osmolytes such as nanoparticle-conjugated trehalose, glucose, and maltose, a class of sugar osmolyte, nanoparticle-conjugated glutamine and histidine, a class of amino acid osmolyte, and nanoparticle-conjugated glycerol, a class of polyol osmolyte, have been already employed to inhibit the various types of therapeutic proteins from aggregation at multiple folds as compared to the molecular forms of osmolytes (Table 1). Many more osmolytes of mixtures of osmolytes need to be functionalized with various types of nanoparticles to elucidate the mechanistic view of protein aggregation.

**Table 1:** Nano-Osmolyte Conjugates and Their Potential to Alleviate Protein Aggregation

entry	nano-osmolyte	nanoparticle size (nm)	mechanism and activity against the protein	type of linkage	mode of denaturation	technique used	protein/cell used for research	enhancement of biological activity (in folds)	ref
1	iron oxide, conjugated to proline/glutamine	20–40	inhibits aggregation of proteins like lysozyme and Huntington protein	covalent linkage between primary amines on the nanoparticle surface and primary amine of l-glutamine	60 °C/24 h	UV–visible spectra, Fourier transform infrared spectroscopy, DLS, ζ-potential, CD, and TEM	lysozyme, HD150Q cell	10^3^–10^4^	(84)
2	carbon conjugated to a sugar osmolyte maltose	20–40	inhibits protein fibrillation and prevents cytotoxicity arising from fibrils	connected with hydroxyl groups	70 °C/24 h for lysozyme, 65 °C for insulin	fluorescence quantum yield, PL and UV–vis spectra, Western blot, and dot plot	Aβ (amyloid β), insulin, lysozyme, HD150Q cell	up to 10^2^–10^4^	(82)
3	Au conjugated to a basic amino acid osmolyte histidine	20–40	completely inhibits fibrillation of amyloid proteins	imine bond	60 °C with the solution at pH 2 in the presence of 140 mM NaCl and 2.5 mM KCl	FTIR, CD, TEM, and DLS	Aβ (amyloid β)	between 10^3^ and 10^4^	(93)
4	highly branched polyglycerol (HPG) dendrimer with gallate, tyrosine, and trehalose	5	enhances bioavailability and multivalent binding with protein	functionalizable surface hydroxyl groups	70 °C/24 h	UV–visible absorption spectra, TEM	lysozyme, HD150Q cell	up to 10^2^–10^3^	(94)
5	carbon conjugated to a sugar osmolyte trehalose	20–40	inhibits protein fibrillation and prevents cytotoxicity arising from fibrils	–OH group	70 °C/24 h	UV–vis spectra, cytotoxicity assay, CD, DLS, and TEM	Aβ (amyloid β), insulin, lysozyme, HD150Q cell	up to 10^4^–10^5^	(82)
6	carbon conjugated to sugar osmolyte glucose	20–40	prevents fibrillation	conjugated with surface −OH groups of glucose	60 °C/24 h	CD, DLS, and TEM	Aβ (amyloid β), insulin, lysozyme, HD150Q cell	∼10^3^–10^4^	(82)
7	Iron oxide polymer conjugated to a sugar osmolyte trehalose	20–30	efficient in brain targeting, entry into neuron cells, and suppression of mutant huntingtin aggregation	conjugated with −OH group	75 °C/7 h	TEM, CD, and UV–vis spectra	Aβ (Amyloid β), Lysozyme, HD150Q cell HD mouse	∼10^3^	(95)

### 6.1 Sugar-Conjugated Nanoparticles

Sugars are osmotically active molecules that accumulate at high concentrations in the cell (for example, trehalose and sucrose) and protect the proteins under stress conditions.^96^ Therefore, trehalose and sucrose are good candidates for preventing protein misfolding and aggregation. However, it is still challenging to administer sugar osmolytes at high concentrations (above the millimolar range) required for their activity. Interestingly, it has been found that conjugated nanoparticles have better chaperonin activity than sugar osmolytes. First, these nanoparticles can be designed with a hydrophobic core of graphitic carbons and the hydrophilic shell of polar sugar molecules so that the conjugated nanoparticles will strongly bind with the misfolded protein to inhibit protein aggregation.^97,98^ Moreover, the multivalent nature of the nanoparticles will interact with more sugar molecules and these multivalent complexes will interact more effectively with the protein moieties to inhibit protein aggregation. Furthermore, nanoparticles with multiple interacting sites can cross the cell membrane very easily via an endocytosis process, which is not possible for molecular sugar.^99^ Thus, enhanced cellular entry of the nanoparticle offers increased interaction with intracellular protein.

Effects of trehalose-based nanoparticles on the fibrillation of human insulin, lysozyme, and amyloid beta (Aβ) have been tested (Table 1).^95,100,101^ It has been found that the trehalose-conjugated nanoparticles can stabilize clinically important proteins in biological environments and can be used as safe formulations without adverse effects *in vivo*. Thus, it shows that the enhanced chaperone performance of sugar-conjugated nanoparticles extends the possibility for practical application (Table 1).

### 6.2 Polyol-Functionalized Nanoparticles

Several polyols including glycerol have been used as green catalysts to reduce metal nanoparticles without using any hazardous chemicals. Due to their unique physicochemical properties, such as high boiling point, low toxicity, ability to form hydrogen bonding, and ability to make both organic and inorganic compounds highly soluble, they act as a good candidates for green chemistry. Genç et al. exploited the reducing ability of glycerol incorporated within nanosized liposomes as a one-pot synthesis to make a homogeneous mixture of gold nanoparticles in the range of 2–8 nm under mild conditions.^102^ Thus, glycerol is a good candidate for the synthesis of controlled and monodisperse size and tunable shape metal nanoparticles for specific applications.

#### 6.2.1 Glycerol Monooleate Nanoparticles (GMO-NPs)

To exploit the polyols to make nanoparticles more biocompatible, glycerol monooleate nanoparticles (GMO-NP) have been used as efficient drug delivery (DD) systems to improve the existing therapeutic strategies. It has been observed that GMO-NP is thermodynamically more stable, nontoxic, and able to encapsulate both types of hydrophilic and hydrophobic drugs and can be administered as a sustained drug-delivery carrier for clinical applications.^103^ In another example, Mandal et al. reported that multiple osmolytes can be used in single-functional dendrimers to stabilize the multiple weak interactions of three-dimensional proteins to prevent the protein from aggregation both intra- and extracellularly. They developed a highly branched polyglycerol dendrimer conjugated with antiamyloidogenic molecules such as tyrosine, trehalose, and gallate and found that this functional dendrimer is more effective in inhibiting protein aggregation at micromolar concentrations as compared to molecular forms of osmolytes (Table 1). Thus, multiple osmolytes can be used as potential nanodrugs for the treatment of various neurological disorders.^94^

### 6.3 Amino Acid Conjugated Nanoparticles

Several bare nanoparticles with various surface chemistry have been reported to induce amyloid aggregation. However, nanoparticles with functionalized amino acids such as histidine have shown promising results against amyloid fibrillation. It has been shown that functionalized nanoparticles with cationic/anionic or hydrophobic groups of amino acids are more effective in modulating the nucleation of protein aggregation (Table 1).^93^ Moreover, amino acids such as proline and glutamine which are effective at molar concentrations as protein stabilizers can be functionalized as multivalent charge nano-osmolytes at the micromolar level and are found to be more effective not only against protein aggregation but also for higher cell uptake. This shows that nano-osmolytes like nanoproline and nanoglutamine can be 1000–10000 times more promising in inhibiting protein aggregation in comparison to molecular forms of amino acids (Table 1).^49,84^

Among multiple amino acid transporters, l-type amino acid transporters (LAT 1) mediate the transport of specific branched and aromatic amino acids across the plasma membrane to feed the nutrients to the metabolic pathways. However, LAT 1 is highly expressed in various types of cancers such as breast, ovarian, and colorectal. Recently, researchers used LAT 1 as a biomarker to detect cancer. In this context, Mathur et al. used tryptophan-conjugated iron oxide nanoparticles by using 3-aminopropyl trimethoxysilane as a linker to target LAT 1 overexpressing tumors and used it as a potential diagnostic kit to locate specific cancers with the help of magnetic resonance imaging (MRI).^104^

#### 6.3.1 Nanoparticles Conjugated with Polymeric Amino Acids

Recently, various types of amino acids and their polymers have been used to conjugate the nanoparticles to enhance the biocompatibility properties, and a more direct synthesis of nanoparticles has been explored to bypass the purification step.^105^ In this regard, Pluronic, an amphiphilic copolymer composed of hydrophilic segments of poly(ethylene oxide) (PEO) and hydrophobic segments of poly(propylene oxide) (PPO) in an “ABA” type (−PEO–PPO–PEO−), has been used efficiently to both reduce and stabilize metallic nanoparticles.^106^ It has been shown that the nanospheres containing lipophilic drugs, synthesized from an amphiphilic copolymer, increased the circulation time in the blood as compared to the free drug and might allow significant multidrug delivery platforms under *in vivo* conditions.^106^

Roy et al.^107^ used cysteine as a reducing and capping agent for AgNP preparation. They found that the prepared AgNPs were stable for a longer period of time and exhibited antimicrobial activity. Csapó and co-workers^108^ synthesized citrate-stabilized AgNPs functionalized with l-cysteine. The antiaggregation-facilitated AgNPs in the presence of osmolyte created a new direction for the colorimetric detection of bioanalytes as well as could be used in the current and forthcoming era with various applications, including therapeutics, cardiovascular implants, dentistry, and biosensors.^109^

## 7 Potential Nano-Osmolyte Aggregation Inhibitors

The chemical chaperone (i.e., osmolyte) conjugates with nanoparticles have already shown promising results in preventing protein aggregations *in vitro* and in cell cultures: for example, the prevention of glycation of alpha-crystallin by conjugation with GNPs.^110^ Similarly, it has also been shown that sugar-based nanoparticles are 100–100000 times more specific and efficient in preventing the fibrillation of proteins and circumvent the cytotoxicity of fibrils as compared to sugar osmolytes.^82^ In another study, polytrehalose–Fe_2_O_3_ conjugates with a size of 20–30 nm were found to be 1000–10000 times more effective in impeding the polyglutamine aggregation in cell and mouse models at micromolar concentrations compared to millimolar and molar concentrations of trehalose.^95^ This result suggests that a nano-osmolyte conjugate can be exploited as a potential candidate for curing protein-aggregation-associated diseases (Table 1).

**Figure 4:**
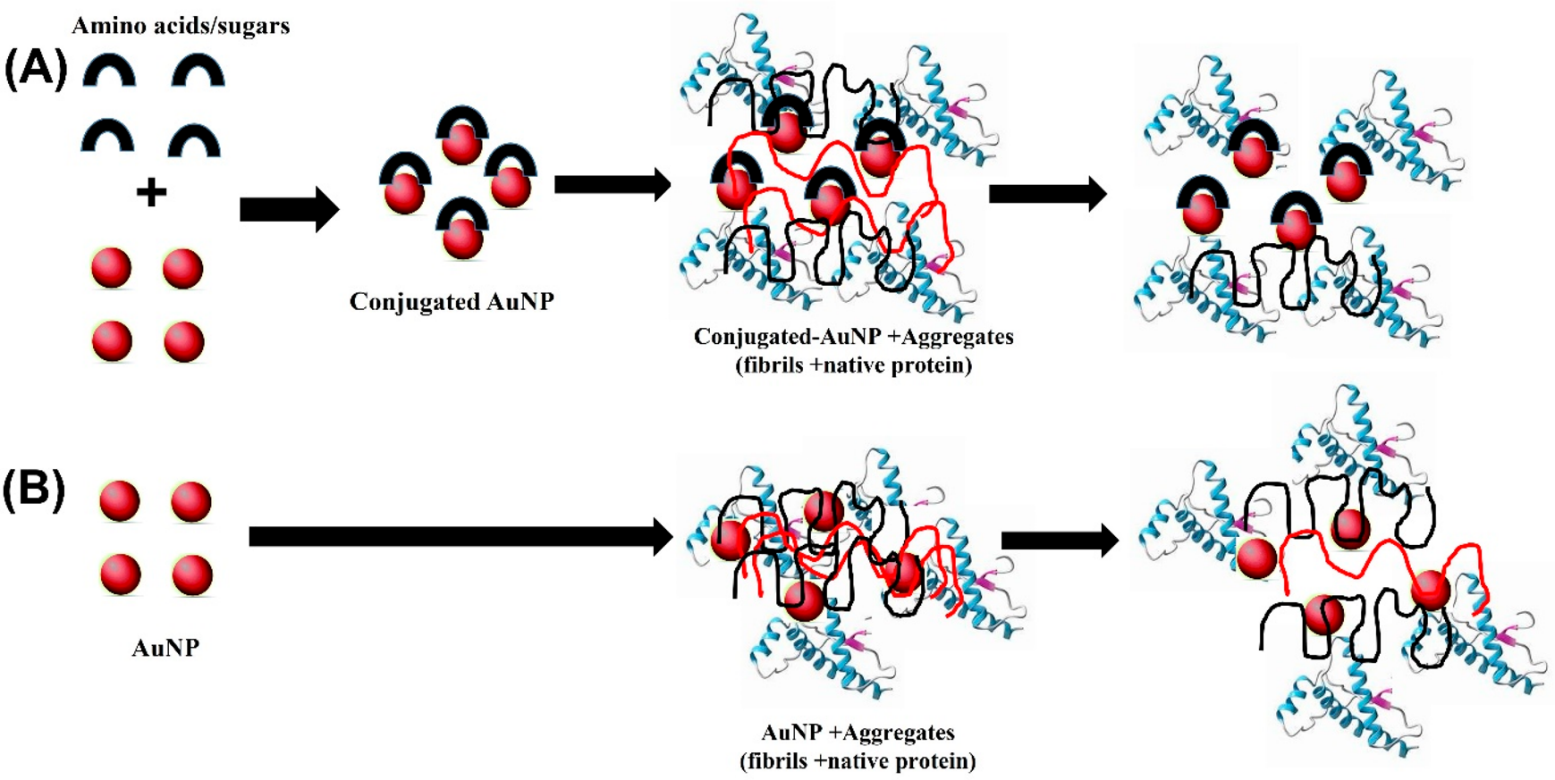
Tentative mechanism for the differential behavior of AuNPs and conjugated AuNPs toward the inhibition of aggregation of the protein. (A) denotes that the conjugation of gold nanoparticles with sugar/amino acid is effective in the inhibition of protein aggregation as compared to the nonconjugated gold nanoparticles as shown in (B).

Among various nanoparticles, AuNPs are considered to have excellent biocompatibility, chemical stability and nontoxic properties and these can be utilized in various biomedical and diagnostic approaches. AuNPs greatly influence the inhibition of protein aggregation and the modulation of the morphology of the protein aggregate.^111^ Moreover, gold nanoparticles with different sizes are effective in preventing different sizes of therapeutic proteins from aggregation at a micromolar concentration.^112^ The surface functionalization of AuNPs with various types of amino acids as nontoxic agents is found to be more biocompatible and can be used as promising nanoparticle conjugates in various biomedical applications.^113,114^ Sen et al. found that the chirality of the amino acid conjugated AuNPs played a vital role in inhibiting protein fibrillation. They showed that d-glutamic acid conjugated AuNP was more effective against forming HSA (human serum albumin) fibrillation than l-glutamic acid conjugated AuNP. This study has depicted that surface chirality acts as a regulator in designing nanoparticles which serve as a control parameter for differential inhibition of the self-assembly of the proteins.^115^Figure 4. shows a possible mechanism of amino acid/sugar conjugated AuNPs that disturbs the nucleation of protein aggregation by adsorbing a large number of fibril/monomers, facilitating the fibrillated protein to gain its native form.^83,84^

Thus, nano-osmolytes show remarkable potential in *in vivo* environments to act as nanochaperones to prevent protein misfolding or act as inhibitors for further binding of monomeric forms of protein for fibrillation (Figure 4).

A major hurdle faced in drug development is the efficient delivery of the drug molecules to the site of action. Delivery of drugs can be achieved much more efficiently with the help of nanoconjugates. For example, ribosome-inactivating proteins (RIPs) such as trichosanthin (TCS) and gelonin (Gel) can efficiently kill cancerous cells, but they are short-lived, are less specific, and have limited efficiency in delivery to the tumor site. To circumvent these problems, a cocarrier of albendazole-encapsulated negatively charged silver nanoparticles with cation-based peptide modified TCS has been utilized. This platform is effective in treating lung tumors.^116^

However, cellular toxicity is a considerable problem associated with drug carriers. For example, metal-based nanoparticles were observed to alter the complement system and increase oxidative stress, which leads to DNA damage and cellular inflammation.^117^ However, amino acids conjugated with liposomes containing drugs interact more effectively with the solute carrier (SLC) proteins to deliver the drugs into the carcinoma cell lines. Moreover, glucose-/galactose-based nanoconjugate formulation enhances the delivery of anti-BACE1 siRNA and 3D6 antibody fragments (3D6-Fab) in the transgenic mouse model of Alzheimer’s disease through the GLUT1-mediated uptake pathway.^118^

Nanoconjugation provides a new direction in the field of protein aggregation. Therefore, nanoconjugation could provide highly efficient therapeutic strategies for protein misfolding diseases. Taking inspiration from the molecular chaperones in maintaining the protein homeostasis of the cell, recent researchers developed an artificial chemical chaperone system consisting of two amphiphilic diblock copolymers which can be used to reduce the Aβ toxicity in the brain.^119^ Similarly, curcumin-based nanoparticles, nanogels consisting of polysaccharide pullulan and cholesterol, and chiral-based penicillamine-capped selenium nanoparticles have been shown to inhibit the fibrillation of Aβ peptides.^120^ Moreover, the liposome-based phosphatidic acid (PA) and APoE complexes have been shown to cross the BBB and disassemble Aβ fibrils in mouse models.^121^

## 8 Conclusion and Perspectives

The unique ability of biocompatibility of osmolytes can be exploited to generate nano-osmolyte conjugates to overcome the pitfalls of osmolytes alone such as nonspecificity, effectiveness at high molar concentrations, and cellular barrier as drug delivery approaches in the treatment of clinical disorders. To enhance the potential effectiveness of osmolytes, the unique physicochemical properties of nanoparticles can be conjugated to make them more effective therapeutically as compared to their molecular forms. For example, nano-osmolyte conjugates have been found to inhibit protein aggregation 10^5^ times better than osmolytes. Understanding the exact mechanism of nano-osmolyte conjugates at a nanomolar concentration against the protein aggregation/protein misfolding shall aid in identifying and designing effective therapeutic nano-osmolyte vis-à-vis their role as nanocarriers for the targeted delivery of drugs. Combinations of nanoparticles with different osmolytes such as hyperbranched polyglycerols, polar osmolytes, and glycerol monooleate nanoparticles are emerging as promising nanoengineered nanodrugs for the prevention of protein misfolding diseases. Several nano-osmolytes with 1000-fold efficiency in preventing protein misfolding and aggregation such as nanoproline, nanoglutamine, and nanotrehalose need to be explored on a larger scale under *in vivo* conditions for their possible transition to clinical trials.

Additionally, a number of other combinations should be explored for the possibility of using them as efficient artificial chaperones. For example, the conjugation of polyols and amino acid derivatives such as trimethylamine *N*-oxide with NPs has not yet been explored to understand the interaction of a conjugated nano-osmolyte with misfolded proteins and treatment of tumors. Furthermore, conjugating multiple osmolytes on the multivalent surface of NPs can be effective in targeting multiple protein receptors on the cell surface and thus might serve as the most efficient and biocompatible drugs for future nanomedicine. In fact, these nano-osmolyte conjugates should be exploited for their formulation as nanochaperone machinery so as to mimic the conventional molecular chaperones for the prevention of newly synthesized proteins from misfolding or acting as antiseeding agents for prevention of protein aggregation/fibrillation.
